# Evaluation of frizzled class receptor 3 and miR-378 expression levels in cumulus cells of polycystic ovary syndrome women: A case-control study

**DOI:** 10.18502/ijrm.v23i3.18777

**Published:** 2025-06-10

**Authors:** Amir Mohammad Bagheri, Kimia Monshizadeh, Fatemeh Anbari, Nasrin Ghasemi, Mohammadreza Dehghani

**Affiliations:** ^1^Department of Medical Genetics, School of Medicine, Shahid Sadoughi University of Medical Sciences, Yazd, Iran.; ^2^Research and Clinical Center for Infertility, Yazd Reproductive Sciences Institute, Shahid Sadoughi University of Medical Sciences, Yazd, Iran.; ^3^Abortion Research Center, Yazd Reproductive Sciences Institute, Shahid Sadoughi University of Medical Sciences, Yazd, Iran.

**Keywords:** Gene expression regulation, Polycystic ovary syndrome, Cumulus cells, Wnt signaling pathway.

## Abstract

**Background:**

Polycystic ovarian syndrome (PCOS) is a common endocrine disorder that affects women of reproductive age. Recent studies suggest that frizzled class receptor 3 (*FZD3*) and miR-378 play significant roles in PCOS by affecting oocyte maturation.

**Objective:**

Considering the importance of *FZD3* and miR-378 in ovulation, the present study aimed to determine the expression levels of *FZD3* and *miR-378* genes in cumulus cells of germinal vesicles and metaphase II oocytes in women with PCOS.

**Materials and Methods:**

The samples for this case-control study included, randomly selected, 25 women with PCOS who were treated at the Research and Clinical Center for Infertility, Yazd, Iran. The diagnosis of PCOS was made based on the criteria defined in the Rotterdam guidelines. Quantitative real-time polymerase chain reaction determined the expression level of *FZD3* and miR-378.

**Results:**

This study showed increased expression of *FZD3* and miR-378 in cumulus cells of immature oocytes compared to mature oocytes (p < 0.0001).

**Conclusion:**

High levels of *FZD3* and miR-378 in cumulus cells of immature oocytes can inhibit their maturation. *FZD3*, a component of the WNT signaling pathway, is overexpressed in immature oocytes and may negatively affect the maturation process. Additionally, miR-378 inhibits oocyte development by targeting and repressing essential genes. Currently, various aspects of microRNA function remain unknown. MiR-378 may exert its regulatory role by directly targeting the *FZD3* gene or by targeting other genes and mediators that interact with *FZD3* or the protein it encodes. This study may provide a foundation for further investigation of this hypothesis in future research.

## 1. Introduction

Polycystic ovarian syndrome (PCOS) is a prevalent endocrine state that affects women of reproductive age (1–3). A complex interplay of hormonal imbalances and metabolic dysregulation characterizes PCOS. Women with PCOS often experience irregular menstrual cycles, polycystic ovaries, and elevated androgen levels (4, 5). The cause of PCOS is unknown, but researchers believe that hereditary factors, diabetes, and, in rare cases, the use of thyroid medication such as methimazole may play a role (6). Given the essential role of genes and microRNAs (miRNAs) in various diseases, it is promising to use them as biomarkers to predict PCOS.

miRNAs are evolutionarily conserved noncoding ribonucleic acids with a length of 18–25 nucleotides (7). miRNAs control the expression of a large part of the human genome (8, 9). miR-378 plays an important role in oocyte maturation (10). One of the most important targets of miR-378 is the enzyme aromatase. This enzyme is involved in the production of estrogen, a key hormone for follicle and oocyte maturation (11). By reducing the expression of aromatase, miR-378 can influence the estrogen level and, thus, the oocyte cell maturation (10).

This microRNA is expressed in cells surrounding the oocyte, such as granulosa cells. It influences the control of oocyte maturation, expansion, and quality by affecting the aromatase enzyme and estradiol product amounts. Changes in the cumulus cells caused by miR-378 can also affect the communication between the oocyte and the surrounding cells and thus influence the maturation process of the oocyte (11). Frizzled class receptor 3 (*FZD3*) is one of the receptors expressed in the cumulus cells (12). Studies have shown that *FZD3* affects the expression of the aromatase enzyme by interfering with the cell's response to follicle-stimulating hormone, leading to changes in the number of estradiol products (13). The steroidogenesis pathway and estradiol products play an important role in the complete and high-quality maturation of oocytes. Therefore, it can be concluded that this receptor plays an important role in the maturation and quality of oocytes. It is also the main site of the secretion of steroid hormones and hormones related to oocyte growth by the cumulus cells. Disruption of steroid function in these cells makes women susceptible to numerous reproductive complications such as menstrual irregularities, follicular immaturity, anovulation, and ultimately infertility. In oocyte development, the germinal vesicle (GV) symbolizes the nucleus of an immature oocyte, indicating that it is still in prophase of meiosis I. As the oocyte matures, the GV degrades, and the oocyte advances to the metaphase II (MII) stage, distinguished by a mature oocyte suitable for fertilization. This transition is essential to proper fertilization and embryo development (14).

Considering the importance of *FZD3* and miR-378 for ovulation, the present study aimed to determine the expression levels of the *FZD3* and *miR-378* genes in the cumulus cells of GV and MII oocytes in women with PCOS.

## 2. Materials and Methods

### Study design

This case-control study examined 25 women with PCOS who were treated at the Research and Clinical Center for Infertility, Yazd, Iran from January to March 2024. The diagnosis of PCOS was made based on the criteria defined in the Rotterdam guidelines (hyperandrogenism, absence of ovulation, and polycystic ovary morphology) (15). The inclusion criteria comprised infertile women with PCOS aged between 30–38 yr, along with cases featuring immature GV-stage oocytes and mature MII-stage oocytes, after evaluating the morphological characteristics of the oocytes. Women with features such as hormonal disorders other than PCOS, infertility from causes other than PCOS, MI-stage oocyte, adrenal gland tumors, congenital adrenal hyperplasia, and Cushing's syndrome were excluded.

### Sample size

Based on a previous study (13) and the sample size calculation technique mentioned below, the sample size for the study was determined to be 25 individuals per group. Therefore, 25 immature oocytes at the GV stage and 25 mature oocytes at the MII stage were used in the study. With a confidence level of 95% and a power of 80%, a standard deviation of 1.2 for *FZD3* gene expression, a mean of 4.2 in cumulus cells of the PCOS group, and a minimum difference of one unit in each group, a sample size of 25 was recommended. The study results and Power Analysis and Sample Size 15 software determined the sample size. 


$$n=\frac{{\left(z_{1}-\frac{\alpha }{2}+z_{1-B}\right)}^{2}\alpha s^{2}}{d^{2}}$$

z1-α/2: The standard normal distribution's crucial value matches the chosen confidence level.

z1-β: The critical value of the standard normal distribution for the desired statistical power.

α\alpha: The significance level, the probability of rejecting the null hypothesis when it is true (type I error).

β\beta: The probability of failing to reject the null hypothesis when it is false (type II error).

s2: The population variance or an estimate of it used to quantify variability in the data.

d: The margin of error or the minimum detectable difference in the parameter of interest.

### Collection and preparation of oocyte-cumulus cell complexes (COCs)

COCs were taken from follicular fluid cases. 5 of these COCs were taken to the research laboratory, the other samples were sent to the treatment department for the therapeutic procedure. The cumulus cells around the oocyte were separated with a special needle. Each oocyte was transferred to a new dish containing G-Mops-V1 medium after washing and placed in an incubator at 37 C and 6% CO ${}_{2}$ for 2 hr. The experiment was performed in a Falcon tube containing 100 μ l of enzyme hyaluronidase at 80 IU/ml concentration from Life Global. Then the tube was placed in the incubator at 37 C for 30 min. The cumulus cells attached to each oocyte were transferred to a Falcon tube and placed in the incubator for 15 min. After 2 washes with 3 ml of phosphate-buffered saline (Inaclon, Iran), the cells were centrifuged at 5000 rpm for 3 min. The resulting pellet was transferred to a 1.5 ml pre-labeled microtube. Subsequently, 250 μ l of RNAlater preservative solution (Thermo Fisher Scientific, Germany) was added to the microtube. The samples were stored in a freezer at -80 C until RNA extraction for analysis of *FZD3* and miR-378 expression.

### RNA isolation and real-time polymerase chain reaction

Following the manufacturer's guidelines, the TRIzol Reagent (Yekta Tajhiz Azuma, Iran) extracted RNA from the cumulus cells surrounding the oocytes. The concentration and quality of the isolated RNA was determined using a spectrophotometer (NanoDrop). The Easy cDNA Synthesis kit (Parstous, Iran) was used to synthesize complementary DNA (cDNA) for *FZD3*.

Subsequently, the quantitative real-time polymerase chain reaction method was used to measure the expression of *FZD3*, for miRNA reverse transcriptase stem-loop, to the miR-378 synthesis designed by AnaCell (AnaCell, Iran). The quantitative real-time polymerase chain reaction method was used to determine the expression of miR-378. Internal controls included Beta-2-microglobulin (*B2M*) (16) and small nucleolar RNA, C/D Box 47 (SNORD47) (17) (Table I).

**Table 1:** The list of primers employed in this research project

**Name**	**Sequence (5 ' → 3 ' )**	
	**Reverse**	**Forward**
*miR-378*	GTGCAGGGTCCGAGGT	ACTGGACTTGGAGTCAGAAGG
*SNORD47*	GTGCAGGGTCCGAGGT	ATCACTGTAAAACCGTTCCA
*FZD3*	GGACAAGGAGGTGAACAAT	GAATGCAGTAGGTTCCCAGA
*B2M*	GCTTACATGTCTCGATCCCAC	GGGTTTCATCCATCCGACATTG
SNORD47: Small nucleolar RNA, C/D Box 47, *FZD3*: Frizzled 3, *B2M*: Beta-2-microglobulin		

### Ethical Considerations

The study design was approved by the Institutional Ethics Committee of Shahid Sadoughi University of Medical Sciences, Yazd, Iran (Code: IR.SSU.MEDICINE.REC.1402.291), and written informed consent was obtained from each participant before sample collection.

### Statistical Analysis

SPSS 26 (SPSS Inc., Chicago, USA) was used for the relevant statistical analysis, and GraphPad Prism (version 8.4.3) to create study diagrams. The *t* test was used to analyze the significance level, the difference in the relative expression of *FZD3* and miR-378 between the GV and MII groups and to compare the demographic characteristics of these 2 groups, and p < 0.05 were considered the significance level.

## 3. Results

In this study, the expression levels of the genes *FZD3* and miR-378 were investigated in cumulus cells of GV compared to MII in PCOS women. In general, the results indicated an increased expression of the genes *FZD3* and miR-378 in the cumulus cells of immature oocytes compared to mature oocytes. The results are listed separately and in detail below.

### Increased *FZD3* gene expression in the GV group compared to the MII group

**Figure 1:**
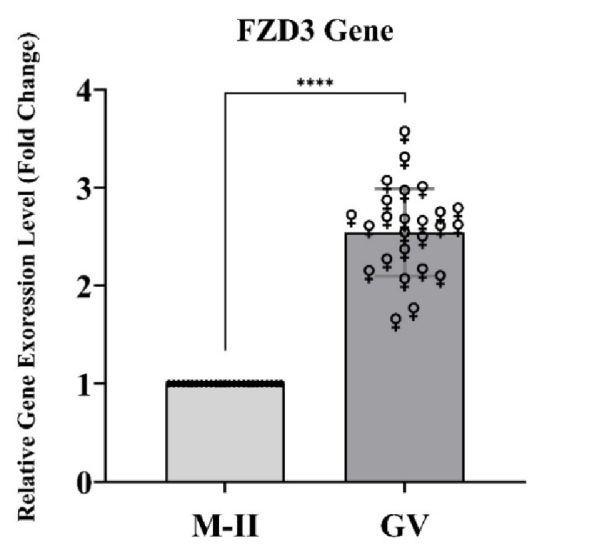
According to the *t* test analysis of the *FZD3* gene, a significant increase in expression (p < 0.0001) was observed in the GV group compared to the MII group. *FZD3*: Frizzled receptor 3, GV: Germinal vesicle, MII: Metaphase II, ****P < 0.0001.

The study's statistical results indicate an increase in *FZD3* gene expression in the GV group compared to the MII group (p < 0.0001). The statistical details of these results are shown below (Figure 1).

### Expression analysis of miR-378

**Figure 2:**
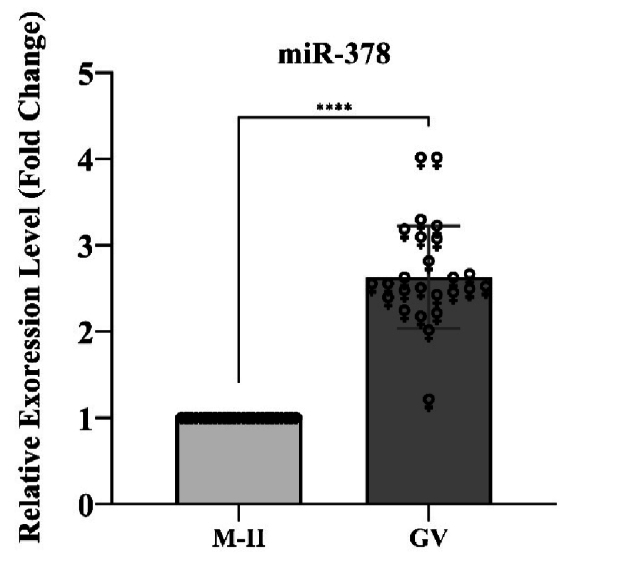
According to the *t* test analysis of the miR-378, a significant increase in expression (p < 0.0001) was observed in the GV group compared to the MII group. ****P < 0.0001. GV: Germinal vesicle, MII: Metaphase II.

Regarding miR-378, the analysis of the results also indicated a significant increase in expression in the GV group compared to the MII group (p < 0.0001). The statistical details of these results are mentioned below (Figure 2).

**Figure 3:**
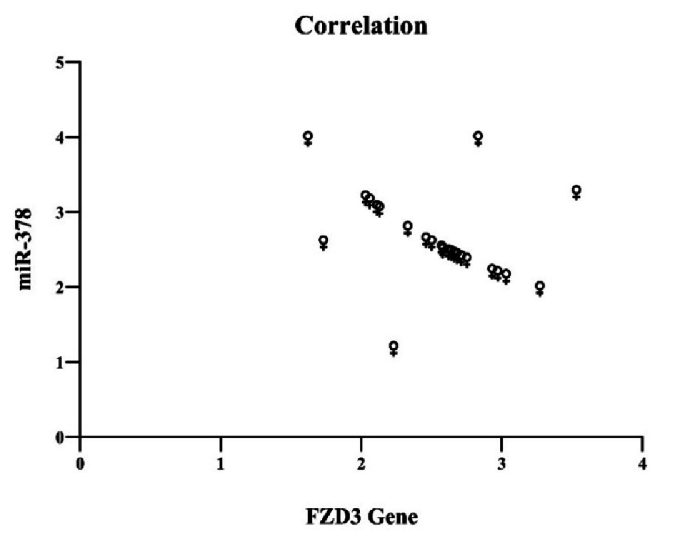
No significant correlation was observed between increased miR-378 expression and *FZD3* gene expression. *FZD3*: Frizzled receptor 3.

Next, the hypothesis was examined through a correlation test to determine whether there was a significant relationship between the increase in miR-378 expression and the *FZD3* gene. The results indicated no significant relationship between these 2 factors' increasing (Figure 3).

## 4. Discussion

This study found increased expression of *FZD3* and miR-378 in cumulus cells of immature oocytes compared to mature oocytes. Thus, the abnormal increase in *FZD3* and *miR-378* gene activity is an obstacle to oocyte maturation. *FZD3* encodes a member of the frizzled gene family (12, 17). The members of this family encode different receptors of the wingless/integrated signaling pathway (WNT). Mutant mouse models and clinical findings indicate that follicular development's most important intraovarian regulators include the tumor growth factor beta/Smad, and WNT/FZD/beta-catenin signaling pathways and their associated components. In addition, the importance of the WNT signaling pathway for ovarian development and oogenesis has been established for many years. Therefore, it appears that any factor associated with the WNT/FZD/beta-catenin signaling pathway could play a role in the developmental course of oocyte maturation (18).

Previous studies have shown that essential WNT pathway genes play important roles in oocyte and embryo development, highlighting their potential as quality indicators. Research using non-human primate and mouse models has shown that members of the *FZD* gene family are both expressed and functional in mature oocytes and embryos. One of the striking findings of this study is that the reduction in expression of these genes during the preimplantation period ensures that components of the WNT signaling pathway function at normal levels.

In contrast, the current study found an aberrant increase in *FZD3* expression in immature oocytes, indicating that the normal downregulation of FZD family genes throughout oocyte maturation and implantation is disturbed. This study agrees with previous conclusions highlighting the relevance of FZD family gene expression throughout oocyte growth and maturation, while expanding these observations by revealing a particular aberrant pattern in immature oocytes (19, 20). In this study, the expression of the *FZD3* gene was found to be elevated in the cumulus cells of immature oocytes compared to mature oocytes. This increased expression may contribute to the maintenance of oocyte immaturity. Unlike studies on animal models, this study was done directly on human oocytes, making it more relevant and applicable to human samples, and more confirmation studies are needed.

Compared to those with normal ovarian function, changes in gene expression patterns in the cumulus cells of women with PCOS are linked to differences in oocyte quality. This study's findings align with previous research indicating that changes in the expression of key cumulus genes, such as *FZD3*, may influence oocyte quality and maturation (21). A recent investigation on estrogen production processes in the cumulus cells of PCOS women found considerably higher levels of *FZD3* gene expression in these cells than in a control group. Notably, the study found increased *FZD3* mRNA levels in PCOS cumulus cells, indicating a link with the disorder. Furthermore, the previous study evaluated *FZD3* expression at the protein level, giving additional support for these findings.

The current study found increased expression of *FZD3*, although it specifically focused on immature oocytes rather than examining the broader context of PCOS. While the study does not confirm this finding at the protein level, it suggests a potential link between elevated *FZD3* levels and oocyte immaturity, offering an additional perspective to previous research (13). The findings of this work support previous reports that an aberrant increase in *FZD3* gene expression in ovarian cumulus cells is linked to oocyte immaturity and malfunction. This supports the relationship between increased *FZD3* activity and impaired oocyte development.

Conversely, the impact of gonadotropin-releasing hormone agonists (GnRH) and antagonists, which are widely used in ovarian stimulation cycles, introduces another layer of complexity. These interventions have been shown to influence gene expression in cumulus cells, significantly affecting oocyte quality and survival. The interplay between GnRH modulation and pathways such as those involving *FZD3* remains an area requiring further exploration to fully understand its implications for oocyte maturation (22). Medical treatments, such as GnRH antagonists like cetrotide, have been shown to affect gene expression in cumulus cells, thereby influencing oocyte survival and quality. Studies indicate that cetrotide impacts WNT-related genes in the mouse ovary, with variations depending on the developmental stage. For instance, the expression of *FZD1* is more prominent in developing mouse oocytes and in adult mouse granulosa cells. Cetrotide administration decreases *FZD1* expression in developing ovaries but does not affect the granulosa cells of mature mouse oocytes. In contrast, *FZD4* expression remained unchanged in all groups, demonstrating differential regulation within the *FZD* gene family.

This study found that FZD3 is expressed at higher levels in the cumulus cells of immature oocytes, suggesting a connection to oocyte immaturity. The data demonstrated both similarities and differences in the functions of FZD family members. While previous research has linked lower FZD1 expression to oocyte immaturity, the current findings indicate that increased FZD3 expression is a significant factor. These distinctions may indicate the different roles of FZD family members during oocyte development, highlighting the complexity of their molecular interactions and the need for further research.

miRNAs play a crucial role in regulating both physiological and pathological cellular functions, including those related to ovarian function. They influence granulosa cell processes such as proliferation, apoptosis, and steroidogenesis, all of which are essential for oocyte maturation. Notably, the expression of miR-378 in granulosa cells varies during follicular development and shows an inverse relationship with estradiol levels in follicular fluid. Investigating the role of miRNAs in signaling networks, such as the WNT/FZD pathway, will improve our understanding of oocyte development and associated disorders.

Although there is limited research on miR-378, overexpression in cumulus cells seems to impair oocyte maturation by decreasing the expression of essential growth and maturation genes (23, 24). Previous studies have demonstrated that miR-378 influences oocyte maturation by inhibiting aromatase activity in the cumulus cells of pigs. It has been shown that miR-378 levels are significantly lower in mature oocyte cumulus cells compared to those in immature oocytes. This suggests that miR-378 plays a crucial role in the development of ovarian cumulus cells. In addition, the overexpression of miR-378 in cumulus cells decreased the expression of genes necessary for oocyte development and maturation, consequently hindering the transition of oocytes from the GV to the MII stage (10). This study used correlation testing to show that increasing gene expression and miR-387 are unrelated. Therefore, this gene is not considered a target of miR-387; however, further research is needed to support this finding. In general, providing a detailed analysis of miR-378 is challenging due to the limited available resources. However, when combining the findings of prior studies with the results of this research, it seems that the enhanced activity of miR-378 might inhibit oocyte maturation.

Despite the remarkable results, this study has shortcomings, including a small sample size, failure to assess the protein expression of miR-378 and *FZD3*, and failure to perform a luciferase assay to confirm the association between miR-378 and *FZD3*.

## 5. Conclusion

High levels of *FZD3* and miR-378 in cumulus cells of immature oocytes can inhibit their maturation. *FZD3*, a component of the WNT signaling pathway, is overexpressed in immature oocytes and may negatively affect the maturation process. Additionally, miR-378 inhibits oocyte development by targeting and repressing essential genes. Currently, various aspects of microRNA function remain unknown. MiR-378 may exert its regulatory role by directly targeting the *FZD3* gene or by targeting other genes and mediators that interact with *FZD3* or the protein it encodes. This study may provide a foundation for further investigation of this hypothesis in future research.

##  Data Availability

Not applicable.

##  Author Contributions

A.M. Bagheri performed the main steps of assay, writing the manuscript, and statistical tests. F. Anbari and K. Monshizadeh. Collected the samples and helped to perform RNA extraction and qPCR. N. Ghasemi and M. Dehghani lead the team, monitored, and fixed technical errors during the study. The authors read and approved the final manuscript.

##  Conflict of Interest

The authors declare that there is no conflict of interest.
